# Virus-host protein co-expression networks reveal temporal organization and strategies of viral infection

**DOI:** 10.1016/j.isci.2023.108475

**Published:** 2023-11-16

**Authors:** Jacobo Aguirre, Raúl Guantes

**Affiliations:** 1 Centro de Astrobiología (CAB), CSIC-INTA, ctra. de Ajalvir km 4, 28850 Torrejón de Ardoz, Madrid, Spain; 2 Grupo Interdisciplinar de Sistemas Complejos (GISC), Madrid, Spain; 3 Department of Condensed Matter Physics and Material Science Institute ‘Nicolás Cabrera’, Science Faculty, Universidad Autónoma de Madrid, 28049 Cantoblanco, Madrid, Spain; 4 Condensed Matter Physics Center (IFIMAC), Science Faculty, Universidad Autónoma de Madrid, 28049 Cantoblanco, Madrid, Spain

**Keywords:** Molecular network, Virology, Proteomics

## Abstract

Viral replication is a complex dynamical process involving the global remodeling of the host cellular machinery across several stages. In this study, we provide a unified view of the virus-host interaction at the proteome level reconstructing protein co-expression networks from quantitative temporal data of four large DNA viruses. We take advantage of a formal framework, the theory of competing networks, to describe the viral infection as a dynamical system taking place on a network of networks where perturbations induced by viral proteins spread to hijack the host proteome for the virus benefit. Our methodology demonstrates how the viral replication cycle can be effectively examined as a complex interaction between protein networks, providing useful insights into the viral and host’s temporal organization and strategies, key protein nodes targeted by the virus and dynamical bottlenecks during the course of the infection.

## Highlights

• Virus-host co-expression networks are reconstructed from temporal proteomics data
• Viral replication cycles are dynamically organized on a network of networks
• The theory of competing networks unveils viral infection complex dynamics
• Network structure and node importance reveal biological functionality

## Graphical abstract

**Figure:**
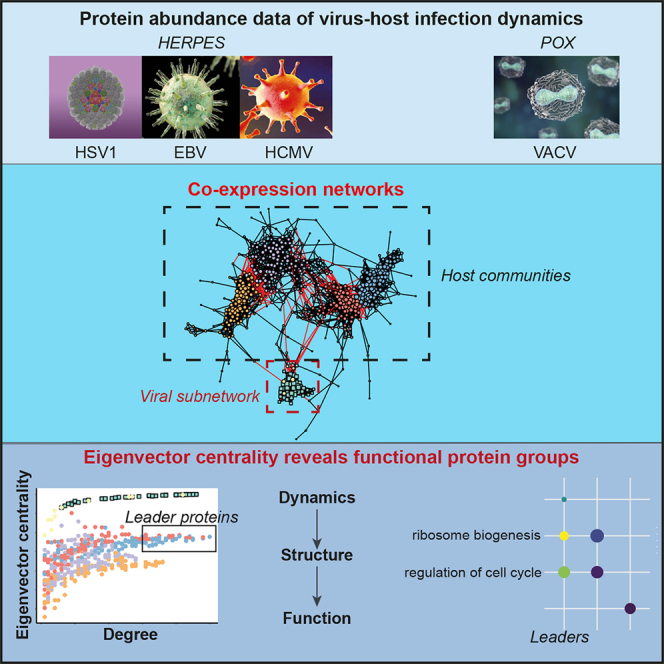

## Introduction

Throughout an infection, viruses hijack different cellular processes of the host cell, dynamically remodeling the abundance, modifications, and interactions of many proteins. Recent improvements in proteomic techniques have enabled precise quantification of thousands of host and viral proteins at different times post-infection.^1^ By simultaneously measuring the abundance of host and viral proteins in a reference (non-infected) situation, these experiments open the possibility to examine which proteins are significantly altered during the infection and when, thus highlighting biologically relevant pathways and potential therapeutic targets.^1^^,^^2^^,^^3^

Currently, most analyses of time-resolved proteome abundances have focused on clustering proteins by similarity in expression profiles, followed by functional analysis such as pathway or gene set enrichment analysis (GSEA).^1^^,^^4^ These studies have revealed host signaling and metabolic pathways affected by the viral infection^2^^,^^5^^,^^6^ and unveiled viral strategies to evade the immune response of the host cell.^3^^,^^7^^,^^8^^,^^9^ On the other hand, many works have addressed the characterization of virus-host molecular interactions from a network perspective, reconstructing the protein interaction networks (PINs) for different pathogens.^10^^,^^11^^,^^12^^,^^13^^,^^14^^,^^15^^,^^16^^,^^17^^,^^18^ This work has uncovered common structural trends in virus-host PINs,^15^^,^^18^ such as that viral proteins tend to interact with densely connected host proteins (hubs).^12^^,^^13^ Despite the usefulness of these studies to comparatively establish principles of viral infection mechanisms,^10^^,^^16^ the nodes of these networks include only proteins with direct physical interactions, thus excluding all indirect regulatory interactions such as transcriptional regulation. Furthermore, and especially for large DNA viruses, the progression of the viral infection depends on the establishment of temporally tuned virus-host and virus-virus protein interactions.^19^ With few exceptions,^20^^,^^21^^,^^22^ most virus-host PINs provide only a static picture of the molecular interactome. In summary, these approaches do not take full advantage of the powerful tools recently developed in the framework of the analysis of dynamical processes on networks in interaction.^23^^,^^24^

In this work, we aim to bridge the gap between the systems level perspective of the viral infection process and its dynamics, by reconstructing and investigating topological and dynamical features of virus-host protein co-expression networks. We make use of the theory of competing networks (TCN),^25^^,^^26^^,^^27^^,^^28^ a mathematical framework designed to analyze the dynamical processes that take place on networks of networks^24^, that is, complex networks connected through a limited number of interlinks, in both equilibrium states^26^ (those that have reached an asymptotic dynamics) and out of the equilibrium.^27^ The latter are more difficult to analyze but are of the maximum importance to understand living entities, as life is grounded on many far-from-equilibrium systems that take place at very different scales. In this methodology, network spectral properties (i.e., the eigenvalues and eigenvectors of the adjacency matrices associated with the networks under study^29^) become critical tools to describe the systems’ complex dynamics, and will help us to perform a thorough comparative analysis of the co-expression networks of four large DNA viruses, with different replication cycle durations and mechanisms. We will focus on (i) how the global network structure reflects the division between virus and host temporal organization, revealing a modular rearrangement of the host proteome during the course of the infection, (ii) how dynamics is naturally included in the network structure through the response time of each node, linking topological features to the temporal program triggered by the viral infection to remodel the host cell machinery, and (iii) how the analysis of node importance provides valuable information on key host proteins and biological processes targeted by the virus. Overall, the integrative approach followed in this work offers a novel perspective on the global temporal organization of a viral replication cycle, and can provide useful biological insight on relevant players and mechanisms of viral infection up to now overlooked in more standard analytical approaches.

## Results

### Quantitative temporal proteomics data and viruses analyzed

We collected experimental protein abundances from time-resolved proteome experiments employing tandem mass tags (TMTs) and triple-stage mass spectrometry (MS3), which considerably reduce experimental noise and allow precise quantitation of thousands of viral and host proteins.^1^ In particular, we analyzed protein abundance data from whole cell lysates of four large double-stranded DNA viruses. Three of them belong to the herpes family: herpes simplex virus 1 (HSV-1),^30^ Epstein-Barr virus (EBV),^5^ and human cytomegalovirus (HCMV).^31^ The fourth virus analyzed, vaccinia virus (VACV)^3^ is an archetype of the pox viral family genetically related to variola virus, responsible for smallpox. Although up to 19 different virus-host systems have been studied with temporal proteomic techniques,^1^ we selected these four viruses based on the following criteria: (i) a relatively large genome and a sizable number of quantified viral proteins, (ii) proteome quantification with the same experimental protocol, to facilitate comparisons among viruses, and (iii) at least five experimental time points to allow for reliable identification of co-expression links (STAR methods). We note that analysis of protein expression in these experiments spans a single replication cycle of the virus, which is considerably longer in HCMV and EBV. Details on the viruses and datasets are provided in Table S1 and the key resources table.

### Virus-host protein dynamics described as a process in a network of networks

To provide a global picture of how viral infection impacts the host proteome, we construct co-expression networks from the time series of protein abundances along the life cycles of the viruses. In these networks, each node represents a protein differentially expressed with respect to the non-infected (control) case at least at one time point, and two nodes are connected through a link if their expression patterns change proportionally (or reciprocally) across time. Specifically, original data were re-analyzed to estimate statistically significant differences (false discovery rates, FDR <0.05) in expression of each protein at each time point, relative to the non-infected samples (STAR methods). A protein was picked as a node, if its differential expression was statistically significant and above a given threshold in relative fold-change abundance, at least at one time point (STAR methods, Table S2 and Figure S1A).

To establish links between nodes, we quantify similarity of changes across time for each protein using a robust measure of proportionality, suitable for relative abundance data.^32^^,^^33^^,^^34^ Similarity is given by a *proportionality* or *concordance coefficient* $\rho_{c}\in \left[-1,1\right]$ with an interpretation similar to a correlation coefficient, ranging between −1 (perfect reciprocity) and +1 (perfect proportionality). Two nodes are connected if their concordance coefficient is above a given cut-off in absolute value (STAR methods, Table S2 and Figures S1B–S1D).

**Figure 1:**
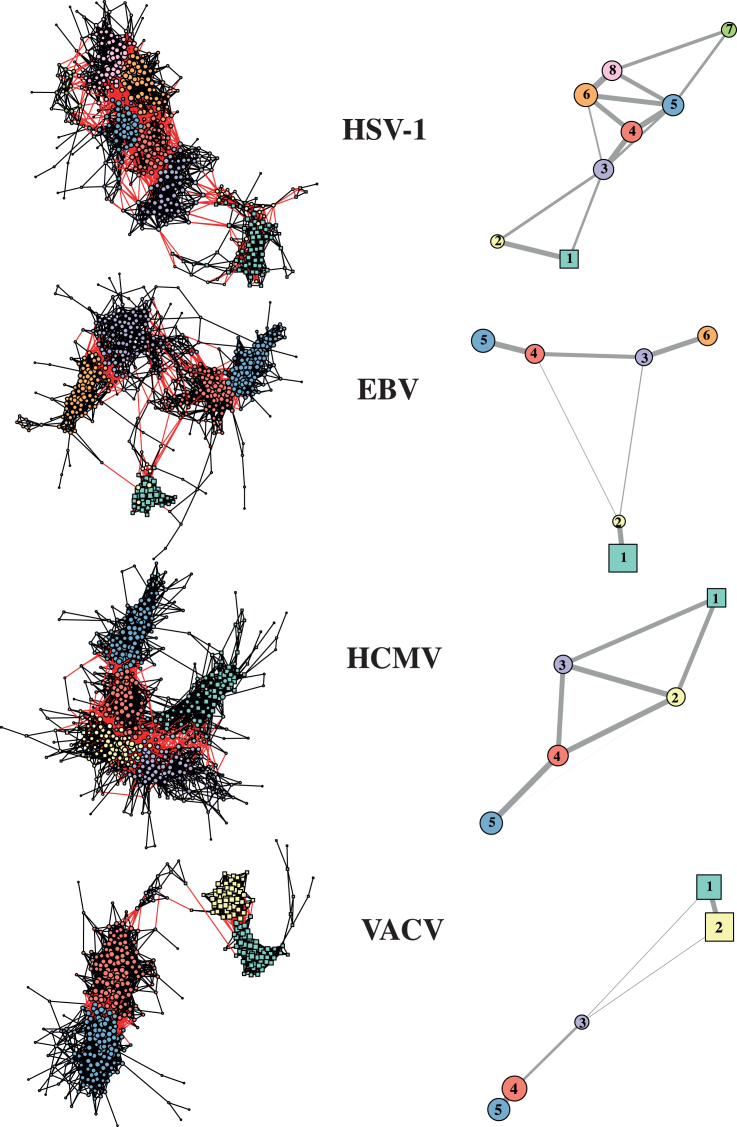
Virus-host protein co-expression networks are modular *Left panels*: Virus-host co-expression networks of four different viruses (Table S1). Nodes correspond to virus/host proteins differentially expressed above a given cut-off value in fold-change abundance with respect to a control (non-infected cells). Links connect nodes changing proportionally (or reciprocally) across time, quantified as a sufficiently high absolute value of a proportionality or concordance coefficient (STAR methods; Tables S1 and S2). Circles: Host proteins. Squares: Viral proteins. Node size is proportional to node degree (i.e., number of neighbors per node), node color denotes community membership and red links are inter-community connections. *Right panels:* Network of communities for each virus, highlighting the modular structure of the co-expression network. Color codes as in left panels. Viral nodes form separate communities (squares) connected to the host network (circles) by one or two small communities, except for the HCMV. Node size is proportional to average internal degree of each community, and edges are weighted with the logarithm of the number of inter-community links.

The co-expression networks obtained with this procedure for all virus-host systems are shown in Figure 1 (left panels). One interesting property of these networks is their modularity, a measure of the strength of division of a network into communities. The modularity coefficient can be either positive or negative, with positive values indicating the presence of community structure.^35^ All virus-host co-expression networks show a modular topology (modularity coefficient > 0.6, Table S2), that makes them behave as networks of networks.^23^^,^^24^ In Figure 1 (right panels) we plot the community network for each virus-host system, that is, a simplified representation of the co-expression networks where each node corresponds to a full community and links connect communities that are interconnected in the whole networks (see Table S3 for the topological description of the different communities).

Importantly, it is evident from Figure 1 that in all co-expression networks virus and host proteins form clearly differentiated subnetworks. In three of them, the activities of viral proteins are so synchronized that they form a single community (two in VACV) of high internal connectivity, i.e., large average degree (average number of links per node). In these systems, the virus-host connection takes place through one or two host communities of low size and average degree (Figures 1 and S3), acting as a weak, but fundamental bridge with the rest of the host network. These *connector communities* are number 2 in HSV-1 and EBV and 3 in VACV (Figure 1 right panels), and they are formed by around 10–20 peripheral host nodes weakly connected among them. Finally, communities are interconnected through *connector nodes* (i.e., those connected to other communities) of a wide range of degree values, but with average degree significantly larger than the *internal nodes* (i.e., nodes that only show connections to their own community), as shown in Figure S3. In summary, the topology of the co-expression networks obtained for the infection dynamics of the four viruses under study can be described as a modular structure where virus proteins are clustered in strongly internally connected communities. In three of the four viruses, these viral communities are clearly differentiated from the host proteins via small host connector communities. Moreover, the host communities are interconnected through proteins of relatively large degree, that is, through densely connected nodes.

### Network topology reflects response dynamics

**Figure 2:**
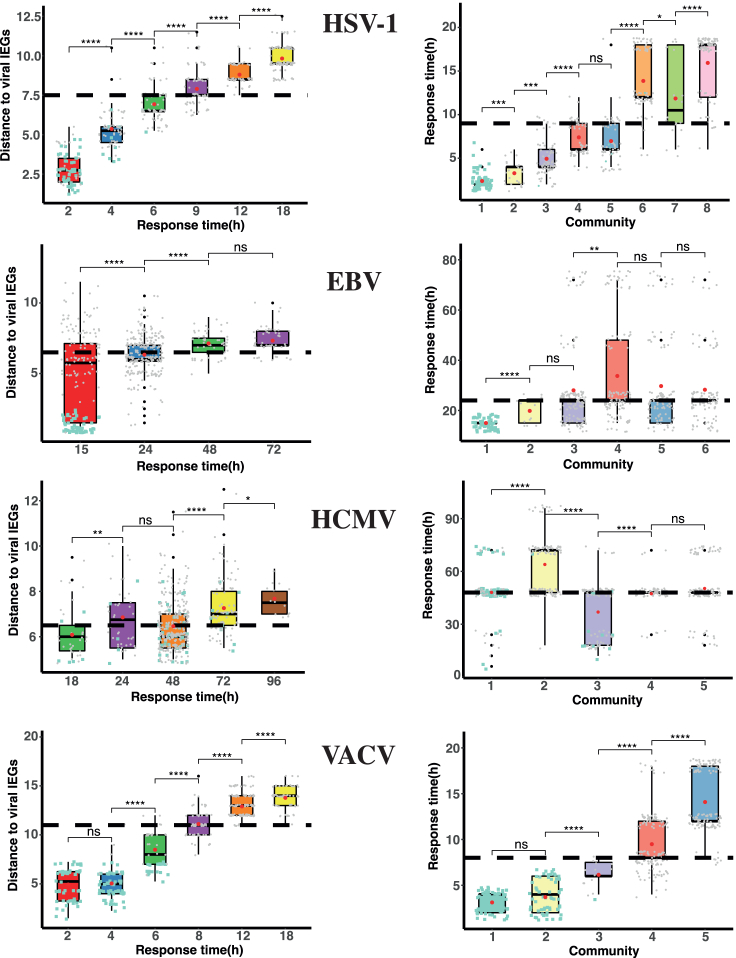
Protein response dynamics at the protein co-expression networks *Left panels*: Distributions (shown as boxplots, red dots are distribution means) of average network distance of each node to viral immediate early genes (IEGs). IEGs correspond to documented viral proteins expressing immediately upon induction of the lytic phase for each virus type, and present in our reconstructed networks (specified in Table S1). Response times are defined as the first experimental time point in which protein abundance exceeds the cut-off in fold-change (STAR methods and Table S2). Box colors are arbitrary and refer to temporal order. Thick dashed lines represent the average distance to IEGs from all nodes in the network. *Right panels:* Distributions (boxplots) of protein response times for the different communities in which each co-expression network is divided. Box colors denote community membership as in Figure 1. Thick dashed lines correspond to the average response times of the networks. In all boxplots, green squares denote viral proteins and gray dots denote host proteins. Stars represent p values for significant differences between distribution means, calculated with two-sided Wilcoxon rank-sum test (ns: p > 0.05; ∗: p ≤ 0.05, ∗∗: p ≤ 0.01; ∗∗∗: p ≤ 0.001; ∗∗∗∗: p ≤ 0.0001).

The co-expression networks associated with the virus-host interaction described here are static, but reflect reliable information about the dynamical process associated with each viral replication cycle. To see this, we assigned to every protein a single response time as the first time to exceed the cut-off in fold-change abundance (STAR methods and Table S2). Figure 2 (left panels) shows the correspondence between the response time of every protein and its network distance (length of shortest path) to the viral immediate-early genes (IEGs, Table S1). In HSV-1, EBV, and VACV, the viral proteins are the ones to be firstly activated, and a “response wave” spreads over the network, starting on the viral communities and advancing toward the furthest host proteins when time increases. Note that the dispersion in the EBV has reached most of the host at 24 h, and therefore the correlation between response time and the distance to the firstly activated proteins is still detectable but less steep (a finer temporal resolution for EBV would be needed to improve these results). In contrast to the other viruses, the HCMV viral proteins (pale blue squares in Figure 2) show a slow response pattern (≥48 h for most of them). This behavior results in a two-phase complex pattern of dispersion over the network, one starting in the first steps of the infection, and the second one beginning around the outburst at 48 h. This pattern reflects the actual life cycle of the HCMV, which is long and dynamically very complex, involving both nuclear and cytoplasmic phases.^36^^,^^37^

The detection of response waves over the networks enables us to analyze the virus-host interaction as a dynamical system taking place on the co-expression network, where relevant perturbations created by the viral machinery spread over the host cells in an organized manner. The right panels in Figure 2 show the relationship between response times and the different communities in which each network is divided. In HSV-1 and VACV the correspondence is clear, implying that the perturbation caused by the infection in the host proteome spreads sequentially over the different communities. Furthermore, note that in these two cases the spreading follows strictly the connection patterns among communities (Figures 1 and 2, right panels). Regarding EBV, and in agreement with the response time-distance correlation studied previously, the perturbation has reached most communities in the first stages of the infection, and only a slightly later response of community 4 is detected. Finally, the HCMV network is more difficult to analyze due to the extensive cross-talk between viral and host proteins,^31^^,^^38^^,^^39^ resulting in strong connectivity between communities (Figure 1 right panel) and slow response dynamics of viral and host nodes. Community 3 responds sooner on average, likely because it contains several early rising viral proteins driving faster changes in the host, which spread later to communities 4 and 5. Community 1, on the other hand, contains many late-rising viral proteins activating the host community 2 in a second stage of the infection process (Figure 2).

### The theory of competing networks unveils infection complex dynamics

The co-expression networks that we are studying here can be considered as networks of networks, where the virus communities interact with very modular co-expression host networks through a relatively small number of interlinks. While the links in each of these networks represent a complex mixture of biochemical and gene regulatory interactions, many of them unknown, and not a clearly defined process spreading on a physical structure (as it could happen in the dispersion of an illness on two populations that get in contact, for example), the systems can be described as response waves that start in the viral communities and sequentially spread over the whole host networks.

Extensive work has been developed on the last two decades to describe the nature of complex interactions in networks of networks and the processes that take place on them, and today it is clear that some of their properties drastically differ from those of dynamical systems spreading on isolated networks, as it arose studying network robustness^40^ or synchronization,^41^^,^^42^ to cite just a few examples. The theory of competing networks^25^^,^^26^^,^^27^^,^^28^ (TCN from now on) approaches the analysis of dynamical processes evolving on networks of networks as a competition for a topological measure called eigenvector centrality, which quantifies the importance of a node taking into account the relevance of its neighbors.^29^ This theory establishes that the spectral properties (eigenvalues and eigenvectors) of the adjacency matrix of the networks under study determine the systems’ complex dynamics (STAR methods). The maximum eigenvalue of the adjacency matrix λ_1_ is a proxy for the strength of a network, and its associated eigenvector has been used in many different contexts with the name of eigenvector centrality to measure the dynamical importance of nodes in a network (or a whole network in a network of networks if we add up the centrality of each network’s nodes).^29^ Some applications of the eigenvector centrality to very diverse contexts are the web page ranking in the internet through the PageRank algorithm,^43^ the study of the impact of a scientist’s work in the community,^44^ the evolution of genotypes,^28^^,^^45^ or the spread of knowledge, innovation, culture, wealth, rumors, or diseases on social groups.^29^ From this perspective, dynamical processes on interconnected networks are analyzed in the TCN as a competition between networks for eigenvector centrality representing importance (or influence, resources, wealth, or whatever the process is describing). The main conclusions of this theory for out-of-equilibrium systems are (i) the system’s dynamics depends (almost) exclusively on the maximum eigenvalues of the different interconnected networks and the eigenvector centrality of the connector nodes, and (ii) the dynamics is fast when networks are strongly interconnected, especially if this is through connector nodes of large internal eigenvector centrality, while few and peripheral inter-network links (i.e., those that link connector nodes of low eigenvector centrality) act as bottlenecks and drastically slow down the dynamics taking place on the system.

**Figure 3:**
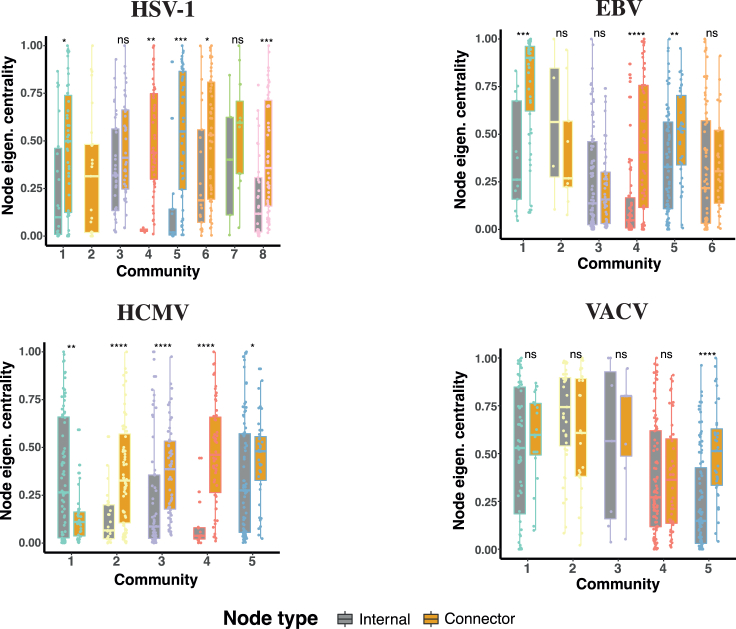
Eigenvector centrality of internal nodes and connector nodes Distributions of node eigenvector centralities for each virus type and community, separating internal nodes (nodes connected only to other members of the same community, gray boxes) from connector nodes (nodes connecting to other communities, orange boxes). Eigenvector centralities correspond to the components (one for each node) of the eigenvector associated with the largest eigenvalue of the network adjacency matrix (STAR methods). Colors of dots denote community membership as in Figures 1 and 2 left. Significance p values for difference of the mean between internal and connector nodes are calculated with two-sided Wilcoxon rank-sum test (ns: p > 0.05; ∗: p ≤ 0.05, ∗∗: p ≤ 0.01; ∗∗∗: p ≤ 0.001; ∗∗∗∗: p ≤ 0.0001).

Let us analyze how the theory of competing networks can be used to describe the dynamics of the virus-host interaction. Table S3 presents the spectral properties of the adjacency matrix associated with the virus network, the host network, and each of the communities in which the co-expression networks have been divided. The distribution of eigenvector centrality of internal nodes (nodes connected only to other members of the same community) and connector nodes (nodes connecting to other communities) of each community in Figure 3 shows that host communities are interconnected through nodes that, on average, have larger centrality than their internal nodes. From the perspective of the TCN, the connection patterns of HSV1, EBV, and VACV suggest a dynamic view in which the perturbation originated by the virus first affects a small number of proteins (the connector communities), acting as dynamic bottlenecks between the virus and the rest of the host. In a second stage, and due to the interconnection between communities through important nodes, the perturbation spreads easily throughout the rest of the host. Furthermore, the drastic imbalance between the centrality accumulated in the virus and the host network (Table S3) is a proof of the isolation of the virus network with respect to the host, while the much more balanced centrality spreading among the different communities of the host networks shows that, while they are clearly topologically differentiated, are nonetheless efficiently interconnected. Note, finally, that in the case of EBV, the viral proteins are so strongly internally connected that its spectral gap (the difference between the two largest eigenvalues, λ_1_−λ_2_) is much larger than the rest of viral networks (a typical property of cliques, or networks where every two nodes are linked). This is due to the fact that the EBV viral proteins behave as an early activated, influential, and highly synchronized cluster.

### Node centrality reveals functional structures

Degree centrality measures the importance of a node in a network based on the number of its neighbors, and therefore represents strictly local influence. Eigenvector centrality, on the contrary, measures the importance of a node based on the relevance of such neighbors, and in practice describes the power of a node to affect other nodes in the whole network. Usual measures of modularity that only take into account the topological structure of networks (based on node degree), if applied to networks where dynamics take place, could yield an incomplete description of the system. On the other hand, using eigenvector centrality to describe the structure of the network can provide a community partition that reveals important dynamical features.^46^

**Figure 4:**
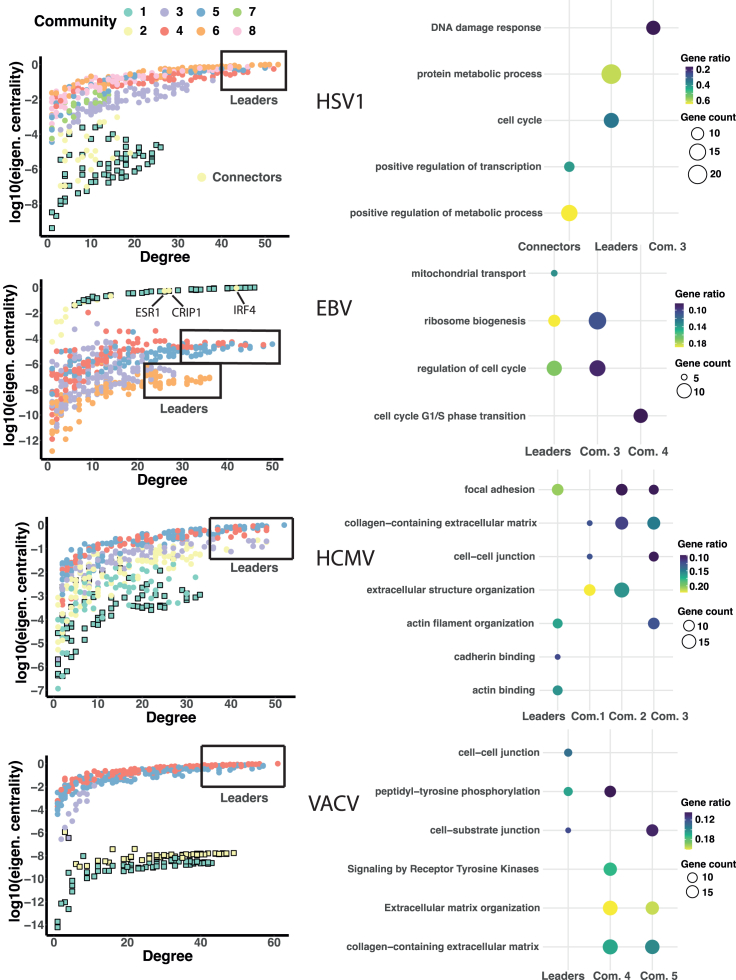
Node centrality and functionality in community structure *Left panels:* Degree versus eigenvector centrality of network nodes. Colors correspond to communities as in Figures 1, 2, and 3. Square filled symbols are viral proteins, circles are host proteins. *Right panels*: Functional analysis of leader nodes, virus-host connector nodes, and communities highlighting the most significant biological processes hijacked by the different viruses based on Gene Ontology, KEGG, and Reactome pathway annotations (STAR methods). Gene ratios (color scale) are the fraction of proteins associated with the particular function within the group analyzed, while Gene counts (size scale) are number of proteins associated with the function within the group.

Following this idea, we show in Figure 4 the node degree versus node eigenvector centrality for each virus, with colors representing the community membership for each protein. This combination of the local and global influence of each protein/node allows us to check, regarding the node clusters present in the plot, whether the communities associated with the system and plotted in Figure 1 show some hidden dynamical structures, and detect the most relevant nodes in the infection process. Figure 4 shows a hierarchical structure of “tongues” for the virus-host networks, in which the viral community is neatly separated from the rest, and connected by a small set of nodes (with the exception of HCMV) to the larger host cluster. The host communities shown in Figure 1 appear as successive layers in the degree/eigenvector centrality plots. In the case of EBV, for instance, two subclusters are clearly distinguished, corresponding to communities 3/6 and 4/5 (see also Figure 1 left).

Next, we explore whether the community and dynamical partitions in Figure 4 reveal preferential functional roles within the host proteome, performing over-representation analysis (ORA) of biological functions based on Gene Ontology, the Kyoto Encyclopedia of Genes and Genomes (KEGG), and Reactome pathway annotations (STAR methods). We focus on communities (especially virus-host connector communities) and on *leader proteins*: those that combine large degree (known as hubs) and large eigenvector centrality (known as central nodes), enclosed within black boxes in Figure 4. In the following, we discuss the main findings in each of the virus-host systems studied (Figure 4 right) and relate them to the known biology of the particular viral infection process.

#### HSV1

One salient topological feature of the HSV1 virus-host network is the presence of a sizable community of connector nodes between the viral and host subnetworks (Figure 4 left). Functional analysis shows that ∼65% of the proteins in this community (all downregulated) are related to positive regulation of cellular metabolism (Figure 4 right), and many of them are transcription factors involved in the regulation of metabolic processes and protein degradation (Table S4). One key mechanism used by HSV1 to escape immunity and promote productive infection is to hijack the ubiquitin-proteasome system,^47^^,^^48^ the main pathway for degradation and functional modification of proteins, orchestrated by the immediate-early viral gene *ICP0*.^49^ Notably, the connector community contains the proteins most sharply downregulated after viral replication induction^30^ (Figure S4A, yellow time series). Several of these connector proteins, involved in the ubiquitin-proteasome pathway, are known to be targeted for degradation by *ICP0*^49^ (Table S4).

Leader proteins (those with high degree and eigenvector centrality, Figure 4 left) play significant roles in the regulation of the cell cycle and in protein degradation (Figure 4 right and Table S4). *ICP0* is known to dysregulate cell cycle, inducing arrest to enhance viral replication.^50^ The alteration of the ubiquitin-proteasome system is also significantly detected in some communities, especially in community 3 and the smaller community 7 (Table S4). Community 3 is also rich in proteins of the DNA damage response pathway (Figure 4 right and Table S4). Again, this pathway is inhibited upon HSV1 infection to favor virus survival, mainly in an *ICP0* dependent manner.^48^^,^^49^

#### EBV

The EBV co-expression network has a small set of virus-host connector nodes (all assigned to community 2, Figure 1). Several upregulated proteins of this community are associated with the complement system,^5^ whose alteration may be connected to the tight association between EBV infection and multiple sclerosis.^51^^,^^52^ The most salient topological feature of the connector community, however, is the presence of three host proteins densely connected to the viral subnetwork (Figure 4 left): interferon regulatory factor 4 (IRF4), which is strongly upregulated, CRIP1 and ESR1. IRF4 has been shown to facilitate EBV lytic reactivation^53^ and is implicated in the differentiation of B cells toward plasma cells associated with many human lymphoid malignancies.^54^ CRIP1, on the other hand, is strongly downregulated and has been identified as a biomarker of EBV-associated nasopharyngeal carcinoma.^55^ The estrogen receptor-1 protein ESR1 (upregulated), is known to induce the expression of the immediate-early viral gene *BZLF1* and has been also associated to poor prognosis of nasopharyngeal carcinoma.^56^

A second distinctive topological feature of the EBV network is the existence of two clusters of communities (communities 4/5 and 3/6, Figure 1) both connected to the viral subnetwork through the connector community 2. This structure is apparent in the degree/eigenvector centrality plots, Figure 4 left, as two “tongues” containing the most connected and central nodes of both clusters. The leader proteins of communities 3/6 are enriched in proteins involved in ribosome biogenesis, all downregulated. The translational machinery of the host cell is modified by different viruses to selectively favor viral replication, and EBV is known to interfere with ribosomal host proteins to activate specific viral genes.^57^ Leaders of communities 4/5 contain especially proteins involved in cell cycle regulation. Like other herpes viruses, EBV induces cell-cycle arrest at G1/S-phase to promote viral replication.^58^ Signatures of this viral reprogramming are noticeable in communities 3 and 4, which connect the two large clusters (Figure 4 right and Table S4).

#### HCMV

The life cycle of HCMV is dynamically very complex, with different groups of viral proteins temporally controlled to be expressed at specific phases of replication.^8^^,^^38^ Time dependent transcriptome and proteome analyses have revealed up to seven different temporal classes,^31^^,^^59^ with some viral genes expressed as late as 3–4 days after the onset of productive infection. This complexity is apparent in the virus-host co-expression network, since viral proteins do not form a clearly independent community from the host as in the other viruses (Figure 4 and Table S3), highlighting the intricate modulation of the host cell proteome during the course of HCMV replication.^36^

The most prominent biological feature throughout the network is a large and significant hallmark of altered host genes involved in focal adhesion as well as in the reorganization of the extracellular matrix and the cytoskeleton. Leader proteins are all downregulated, and mostly involved in focal adhesion and actin filament organization (mainly through cadherin and acting binding, Figure 4 right). Notably, one of the most connected proteins is adducin 3 (ADD3), involved in the assembly of the actin filament network at sites of cell-cell contact. This gene is a marker of biliary atresia,^60^ a pathology also associated to HCMV infection.^61^ Functions related to adhesion and extracellular matrix organization are also over-represented in the rest of the communities, consistent with a global downregulation of these processes in HCMV infected cells.^62^ The signatures observed here are also in agreement with a general reprogramming of mesenchymal-to-epithelial (MET) and epithelial-to-mesenchymal (EMT) transition pathways recently uncovered in HCMV infection.^63^ Dysregulation of these pathways, that are associated to cancer progression and prognosis,^64^ is temporally organized across different communities (Table S4).

#### VACV

Poxviruses are characterized by conducting the whole infectious cycle within the cytoplasm of the host cell, without direct involvement of the nucleus. As an archetype of this viral family, vaccinia encodes virtually all the proteins necessary for replication in the cytoplasm, which takes place in “replication factories” that compartmentalize and help to protect the viral genome from immune detection. The low interconnectedness observed between viral and host communities for VACV likely reflects this autonomous replication mechanism. The distinctive feature of the VACV co-expression network is the existence of two separated viral communities (communities 1 and 2, Figure 1), corresponding to two well differentiated time profiles (Figure S4D, green and yellow time series). Community 1, which includes the viral nodes most sharply activated upon infection, contains the proteins initially involved in the core replication machinery^65^ (Table S4). On the other hand, the nodes belonging to the viral community 2 activate in a more graded manner and at later times. This community includes the proteins involved in different viral phases, from immature virion assembly to envelope formation,^66^ Table S4. The viral subnetwork partition thus reflects the dynamic organization of the VACV life cycle.

There are around 50 leader proteins, all downregulated, belonging to communities 4 and 5, Figure 4 left. These include many plasma membrane proteins, which are globally modulated during VACV infection to evade the immune system and alter the cell host motility.^67^ Of note, many of these are proteins involved in the Ephrin signaling pathway^68^ (Table S4) which is used by different viruses for viral entry.^69^ Ephrins are known to modulate cell adhesion and migration properties.^68^^,^^70^ The strong downregulation of Ephrin receptors observed within the leader community, which could facilitate cell-cell repulsion and increase cell migration,^70^ suggests the mechanism employed by VACV to induce cell motility and enhance the spread of infection.^71^ In agreement with this, we observed a large and highly significant functional role of both receptor tyrosine-kinase mediated signaling (involved in cell adhesion and migration) and extracellular matrix reorganization in the host communities (Figure 4 right).

In summary, the combined topological and functional analyses of our reconstructed co-expression networks highlight the key mechanisms by which different viruses replicate and modulate the host proteome to evade immunity and enhance the spreading of infection. Communities and leader proteins preferentially reflect the alteration of a particular pathway and the organization of its temporal response.

## Discussion

Taking advantage of a complex network perspective, we presented here a thorough systems level analysis of the temporal program of virus and host proteome reorganization after productive viral infection, using quantitative time course data of four large DNA viruses with complex replication cycles. Despite the specific characteristics of the different viruses, the application of the theory of competing networks shows how the infection process can be seen as the interaction between two co-expression networks (the virus and host proteomes) in which the perturbation induced by the virus propagates in a temporally coordinated manner through the host network. Upon lytic activation, HSV1, EBV, and VACV replicate in a relatively autonomous and synchronized manner independent of the dynamics of the host response. This is especially clear in the EBV co-expression network, and consistent with the fact that this virus encodes its own machinery to initiate DNA amplification and to proceed to late gene expression.^72^ Our reconstructed networks for these viruses show that viral proteins are connected to a small set of host proteins of low average degree. This is in contrast to the observed topology in virus-host PINs, in which viral proteins preferentially target host proteins that are densely connected (hubs).^12^^,^^13^^,^^15^ Studies in virus-host PINs suggest that host proteins targeted by viruses are evolutionarily conserved^12^^,^^73^ and can control a wide range of functions. In our case, the weak connections between viral and host subnetworks are the hallmark of a dynamical bottleneck between the onset of viral replication and the propagation of the perturbation to the host proteome. Host connector nodes may functionally represent proteins targeted by viral early genes to establish a host environment favorable for replication, as is the case in HSV1,^49^ or promoting lytic activation, as in EBV infected B cells.^53^

Based on TCN’s predictions, the modularity and topological structure of the host network (where connector nodes between communities have a larger eigenvector centrality on average) suggest a second dynamical phase in which the perturbation produced in a few host proteins propagates more efficiently between communities. Functional analyses of different communities show, in fact, that they do not act as independent functional modules but may be part of the same host pathways remodeled through different temporal stages. For instance, two or more interconnected communities are involved in the dysregulation of cell adhesion and motility properties of the infected cells in both VACV and HCMV. Additional substructure within communities may be revealed by a joint analysis of degree/eigenvector centrality, highlighting groups of important nodes (leader proteins) with relevant functional roles. Since these are proteins that co-vary in time in a concerted way with many other proteins, they can be naturally seen as nodes synchronizing the activity of other nodes involved in similar functions.

The global structural and dynamical analysis of co-expression networks of different virus-host systems as performed here, apart from pinpointing important nodes or sets of nodes targeted by each virus, provides a means to uncover common mechanisms of viral infection. One prominent example is the extensive remodeling of cell adhesion and migration pathways in fibroblast infection by two viruses belonging to different families, HCMV and VACV. Many of the proteins downregulated by VACV infection related to these pathways, such as different collagen types, thrombospondins and metalloproteases, were also downregulated in HCMV infection^3^^,^^67^(Table S4), pointing out to similar viral strategies of these two large viruses to spread infection despite the different life cycles and mechanisms used for replication. Another relevant example highlighted by our analyses is the suppression of the SMC5/SMC6 complex in both HSV1 and EBV infection. In the case of EBV, lytic replication requires the formation of replication compartments in the nucleus.^74^ Notably, the complex SMC5/SMC6, strongly downregulated in community 3, acts as an immune sensor and restricts the formation of replication compartments. This complex has been shown to be directly targeted for degradation by the EBV viral protein BNRF1.^9^ In HSV1, the SMC5/SMC6 complex is downregulated in community 7 (Table S4). This complex can also act as a SUMO ligase promoting DNA synthesis and repair,^75^ consistent with the alteration of the DNA damage response pathway induced by HSV1.^48^ These observations confirm the role of the SMC5/SMC6 complex as an important node of the host defense mechanism.^76^

Finally, the co-expression networks reconstructed here are based on a parsimonious procedure and a sensible measure of proportionality. Many different methods exist to infer co-expression networks, mainly in the context of gene transcription and many experimental samples.^77^^,^^78^^,^^79^ Although other methods could generate different network structures and produce different communities, our procedure recovered a connected virus-host network dynamically meaningful. The correspondence between dynamics and network topology was especially clear in the two viruses with shorter life cycles and better temporal resolution, HSV1 and VACV. These results show that the methods used here, combined with the theory of competing networks, can become useful analysis tools as quantitative temporal viromics increases its multiplexing capacity improving temporal resolution.

### Limitations of the study

An important limitation of this kind of studies is the lack of mechanistic explanation for most of the co-expression links obtained (whether they truly represent direct or indirect interactions, physical or regulatory, etc.) Of note, practically all of the host nodes present in the HSV-1 and VACV networks are downregulated (Table S3), consistent with the finding that these two viruses induce degradation of many host proteins by hijacking the ubiquitin-proteasome system.^80^^,^^81^ Nevertheless, many of these downregulated proteins may take place via a proteasome-independent mechanism.^67^

A second limitation of our study is imposed by the nature of the datasets analyzed (whole cell lysates), which cannot capture differences in subcellular localization. In order to facilitate viral entry, escape immunity and promote infection spreading, many viruses cause extensive remodeling of cell surface proteins. Indeed, for the viruses studied here, the effect of viral infection on the plasma membrane proteome has been separately investigated.^5^^,^^30^^,^^31^^,^^67^ Although we did not specifically analyze membrane proteins, we notice that many of the important nodes pinpointed by our analyses, especially in HCMV and VACV (with extensive remodeling of cell adhesion and migration properties) involve proteins localized in the plasma membrane.

## STAR★Methods

### Key resources table

**Table:** 

REAGENT or RESOURCE	SOURCE	IDENTIFIER
**Deposited data**		

Proteomics data of HSV-1 whole cell lysate time course	Soh et al.,^30^ Table S1	https://doi.org/10.1016/j.celrep.2020.108235
Proteomics data of EBV whole cell lysate time course	Ersing et al.,^5^ Table S1	https://doi.org/10.1016/j.celrep.2017.04.062
Proteomics data of HCMV whole cell lysate time course	Nightingale et al.,^38^ Table S1	https://doi.org/10.1016/j.chom.2018.07.011
Proteomics data of VACV whole cell lysate time course	Soday et al.,^3^ Table S1	https://doi.org/10.1016/j.celrep.2019.04.042

**Software and algorithms**		

*edgeR*	Chen et al.^82^	https://bioconductor.org/packages/release/bioc/html/edgeR.html
*poweRlaw*	Gillespie.^83^	https://www.jstatsoft.org/article/view/v064i02
*igraph*	Csardi & Nepusz.^84^	https://igraph.org/
*clusterProfiler 4.0*	Wu et al.^85^	https://bioconductor.org/packages/release/bioc/html/clusterProfiler.html
*gprofiler2*	Kolberg et al.^86^	https://cran.r-project.org/web/packages/gprofiler2/index.html
*GeneCodis 4*	Garcia-Moreno et al.^87^	https://genecodis.genyo.es/
*TMixClust*	Golumbeanu et al.^88^	https://bioconductor.org/packages/release/bioc/html/TMixClust.html

### Resource availability

#### Lead contact

Further information and requests for resources should be directed to and will be fulfilled by the lead contact, Raúl Guantes (raul.guantes@uam.es).

#### Materials availability

This study did not generate new unique reagents.

#### Data and code availability

• All data produced in this study are included in the published article, its supplemental information, or are available from the lead contact upon request.
• This paper does not report original code.
• Any additional information required to reanalyze the data reported in this paper is available from the lead contact upon request.

### Method details

#### Collection and reanalysis of virus-host temporal proteomic data

We gathered protein abundances from time resolved proteome experiments of human cell lines infected with four large double-stranded DNA viruses: Herpes Simplex Virus 1(HSV-1),^30^ Epstein-Barr Virus(EBV),^5^ Human Cytomegalovirus (HCMV)^31^ and Vaccinia virus (VACV).^3^ All original data collected (key resources table) were generated with the same experimental protocol, using tandem mass tags (TMT) for peptide labeling and triple-stage mass spectrometry (MS3), which allows quantification of all samples (time points, controls …) in a single experimental run.^1^ This technique reduces experimental variability and facilitates reliable comparisons across conditions. Original data were reported in pseudo-counts, corresponding to normalized signal-to-noise ratios (values relative to maximal signal observed for each protein in each TMT channel).

In order to extract the most affected proteins during viral life cycles with comparable criteria among viruses, original data were reanalyzed for differential expression with respect to a control (non-infected or uninduced samples) using the *edgeR* pipeline^82^ (key resources table). All viruses analyzed, with the exception of HSV-1, had at least two biological replicates. We used a paired design (mock versus infected samples at each time point) controlling for differences between biological replicates, by fitting a negative binomial generalized log-linear model to protein pseudo-counts (functions *glmQLFit* and *glmQLFTest* in *edgeR*). For samples/time points without biological replicates, we estimated the significance in differential expression using dispersions from the closest time points. For HSV-1, we used the dispersions obtained from the VACV, which was quantified by the same experimental protocol at the same time points. We found that only ∼12–18% of all quantified host proteins were differentially expressed (FDR <0.05) with respect to the non-infected case at least at one time point, but nearly all quantified viral proteins over-expressed with respect to the control (Table S1). To take into account possible effects of composition bias between samples, we used trimmed mean of M-values (TMM) normalization^89^ of data for further analyses, as has been done in other quantitative temporal viromics studies.^2^

#### Reconstruction of virus-host protein co-expression networks

Our goal is to provide a global picture of the most important players and their dynamical relationships in the virus-host infection process. We thus seek to reconstruct a manageable virus-host protein co-expression network in which the nodes are the virus/host proteins most significantly altered during the course of infection (with respect to the non-infected control) and the links between nodes reflect which proteins change more similarly in relative abundance across time.

To choose the nodes of the network, we impose two conditions: that the differential expression of a given protein with respect to the control condition is statistically significant (FDR >0.05), and that the fold-change in abundance (infected-control ratio) is above a given cut-off, that we chose based on abundance/fold-change plots, Figure S1A. Whenever these two conditions are met in at least one time point, we select this protein as a node for network construction.

To establish the links between nodes, we need a quantitative measure of ‘similarity’ in abundance changes across time. Since quantified protein levels are always relative abundances (with respect to total counts of ion fragments), usual measures of correlation are biased.^32^^,^^90^ A better strategy is to interpret co-expression as *proportional* changes across conditions. The idea behind is that if relative abundances of proteins *p*_*i*_ and *p*_*j*_ are proportional across experimental conditions, their absolute abundances must be also in proportion.^32^ Proportions are calculated with a log-ratio scaling of protein abundances, and co-expression between every protein pair is quantified by a *proportionality* or *concordance coefficient* ρ_c_, obtained by estimating the log-ratio variances as^32^^,^^33^:

$$\rho_{c}\left(p_{i},p_{j}\right)=1-\frac{\text{var}\left(p_{i}-p_{j}\right)}{\text{var}\left(p_{i}\right)+\text{var}\left(p_{j}\right)}\text{.}$$

Here, the proportions *p*_*i*_*, p*_*j*_ are calculated from the original protein abundances *x*_*i*_*, x*_*j*_ by a centered log-ratio transformation:

$$p_{i}=\log\left(\frac{x_{i}}{g_{m}\left(x\right)}\right)\text{,}$$

where $g_{m}\left(x\right)$ denotes the geometric mean of all original protein abundances.

The concordance coefficient was first proposed as a reproducibility index of independent measurements^91^ and has the same interpretation as a correlation coefficient, ranging between −1 (perfect reciprocity) and +1 (perfect proportionality). To reconstruct the virus-host co-expression network, we consider that two proteins are linked if the absolute value of their concordance coefficient is above a certain cut-off (Table S2). The cut-off value is chosen based on the distribution of concordance coefficients and the number of nodes/links remaining, to avoid either too sparse/disconnected networks or too dense networks (Figures S1B–S1D). These values are very similar to the cut-offs defined in other applications of proportionality measures used in the analysis of transcriptomic data.^32^^,^^33^^,^^92^ A recent thorough study on the performance of different association methods to reconstruct cellular networks from scRNA-seq data systematically scored proportionality measures as the best performing methods.^34^

#### Network analysis and visualization

All network analyses where done in R programming language, with extensive use of the *igraph* software package (key resources table) for graph visualization and calculation of network properties.

#### Community partition

For community partition and further analyses, we took only the giant component (largest connected component) of the reconstructed network. To identify communities (sets of nodes more densely connected among them than with other sets of nodes) we used the recently developed Leiden algorithm^93^ which is fast and guarantees well-connected communities. Moreover, it provides flexibility in the number of communities defined by changing a resolution parameter γ: higher resolution leads to more communities, while lower resolution results in fewer communities. To select the best community partition for each virus-host network, we applied the Leiden algorithm to optimize modularity in a range of γ values (0.1 < γ < 2). Simultaneously monitoring the number of different communities and the modularity coefficient, we chose the optimal γ value as the one providing the highest modularity and the most robust community partition (Figure S2).

#### Analysis of biological functionality

Functional enrichment or over-representation analysis (ORA) based on Gene Ontology, KEGG and Reactome pathway annotations were conducted for different groups of proteins extracted from the reconstructed co-expression networks (communities, connector nodes and sets of *leader* proteins, Figure 4 left). Analyses were undertaken with different functional annotation tools: *gprofiler2*,^86^ *clusterProfiler* 4.0^85^ and *GeneCodis4*^87^ with default parameter settings. Statistically significant annotation terms consistently appearing under different pipelines were extracted, and the most relevant annotated proteins were manually searched to confirm their biological role. The most significant terms and groups of proteins were represented as dotplots in Figure 4. A complete list of all the protein symbols and names associated with a given function in each group of nodes is provided in Table S4.

#### Eigenvector centrality and the theory of competing networks

A network can be mathematically represented by its adjacency matrix **A**, which for a network of *N* nodes is defined as the N×N matrix with elements *A*_*ij*_ such that *A*_*ij*_ = 1 if nodes *i* and *j* are connected, and 0 otherwise. The eigenvector centrality can be obtained as the eigenvector **u**_1_ associated with the largest eigenvalue λ_1_ of the adjacency matrix of the network under study.^29^ It can be calculated for a single node *i* (as the element *i* of vector **u**_1_), or accumulate it on a set of nodes as the sum of their centralities (normalized such that the total centrality of the whole network is 1). The maximum eigenvalue λ_1_ typically grows with the number of nodes and links of a network, and therefore can be used as a proxy for the strength of a network in a dynamical process where several networks interact. The eigenvector centrality **u**_1_ and the two largest eigenvalues of the system λ_1_ and λ_2_ are directly related to the dynamics as follows: If a process takes place on a network such that **n**(*t*+1) = **M n**(*t*), where **n**(*t*) is the system state at time *t* and **M** is the transition matrix associated with the dynamical system under study, in the limit *t*→∞, **n**(*t*) attains a stationary state independent of the initial condition that coincides with the eigenvector centrality **u**_1_, and the maximum eigenvalue λ_1_ yields the growth rate of the population at such stationary state. Furthermore, the time to equilibrium is proportional to ln(λ_1_/λ_2_)^−1^.^45^

The dynamical processes that take place on interconnected networks can be analyzed in the context of the TCN^25^^,^^26^^,^^27^ as a zero-sum competition between networks for importance/centrality, measured as the accumulated eigenvector centrality in the nodes of each competitor. An explicit expression can be obtained for the outcome of the competition for centrality between several networks, as well as the time to equilibrium in such competition.^25^ A summary of the main results of the TCN is: (1) the outcome of the competition for centrality between two interconnected networks is only dependent on the largest eigenvalue of the adjacency matrix of each network in the system and on the eigenvector centrality of the connector nodes; (2) increasing the number of interlinks and/or the centrality of the connector nodes of the two competing networks accelerates the dynamical process and increases the final centrality of the weak network, while a low number of interlinks that only connect peripheral nodes will give rise to a very slow process that strongly increases the final centrality of the strong network.

### Quantification and statistical analysis

Statistical significance of differences between means of the distributions shown in Figures 2 and 3 have been calculated with the *compare_means* function in R, using the two-sided Wilcoxon sum rank test, as specified in the Figure legends.

False discovery rates for significance of differential expression were calculated by the *edgeR* pipeline (key resources table) using the *glmQLFTest* and *topTags* functions. Other quantification procedures are described in the ‘method details’ section above.

## Supplemental information

Document S1. Figures S1–S4 and Tables S1–S3

Table S4. Protein symbols and approved names corresponding to the significantly enriched functions shown in Figure 4, for each virus

## References

[1] Fletcher-Etherington A., Weekes M.P. (2021). Quantitative Temporal Viromics. Annu. Rev. Virol..

[2] Bojkova D., Klann K., Koch B., Widera M., Krause D., Ciesek S., Cinatl J., Münch C. (2020). Proteomics of SARS-CoV-2-infected host cells reveals therapy targets. Nature.

[3] Soday L., Lu Y., Albarnaz J.D., Davies C.T.R., Antrobus R., Smith G.L., Weekes M.P. (2019). Quantitative Temporal Proteomic Analysis of Vaccinia Virus Infection Reveals Regulation of Histone Deacetylases by an Interferon Antagonist. Cell Rep..

[4] Rahmatbakhsh M., Gagarinova A., Babu M. (2021). Bioinformatic Analysis of Temporal and Spatial Proteome Alternations During Infections. Front. Genet..

[5] Ersing I., Nobre L., Wang L.W., Soday L., Ma Y., Paulo J.A., Narita Y., Ashbaugh C.W., Jiang C., Grayson N.E. (2017). A Temporal Proteomic Map of Epstein-Barr Virus Lytic Replication in B Cells. Cell Rep..

[6] Wang L.W., Shen H., Nobre L., Ersing I., Paulo J.A., Trudeau S., Wang Z., Smith N.A., Ma Y., Reinstadler B. (2019). Epstein-Barr-Virus-Induced One-Carbon Metabolism Drives B Cell Transformation. Cell Metab..

[7] Greenwood E.J., Matheson N.J., Wals K., van den Boomen D.J., Antrobus R., Williamson J.C., Lehner P.J. (2016). Temporal proteomic analysis of HIV infection reveals remodelling of the host phosphoproteome by lentiviral Vif variants. Elife.

[8] Nightingale K., Potts M., Hunter L.M., Fielding C.A., Zerbe C.M., Fletcher-Etherington A., Nobre L., Wang E.C.Y., Strang B.L., Houghton J.W. (2022). Human cytomegalovirus protein RL1 degrades the antiviral factor SLFN11 via recruitment of the CRL4 E3 ubiquitin ligase complex. Proc. Natl. Acad. Sci. USA.

[9] Yiu S.P.T., Guo R., Zerbe C., Weekes M.P., Gewurz B.E. (2022). Epstein-Barr virus BNRF1 destabilizes SMC5/6 cohesin complexes to evade its restriction of replication compartments. Cell Rep..

[10] Bösl K., Ianevski A., Than T.T., Andersen P.I., Kuivanen S., Teppor M., Zusinaite E., Dumpis U., Vitkauskiene A., Cox R.J. (2019). Common Nodes of Virus–Host Interaction Revealed Through an Integrated Network Analysis. Front. Immunol..

[11] Brito A.F., Pinney J.W. (2017). Protein–Protein Interactions in Virus–Host Systems. Front. Microbiol..

[12] Calderwood M.A., Venkatesan K., Xing L., Chase M.R., Vazquez A., Holthaus A.M., Ewence A.E., Li N., Hirozane-Kishikawa T., Hill D.E. (2007). Epstein–Barr virus and virus human protein interaction maps. Proc. Natl. Acad. Sci. USA.

[13] Dyer M.D., Murali T.M., Sobral B.W. (2008). The Landscape of Human Proteins Interacting with Viruses and Other Pathogens. PLoS Pathog..

[14] Gordon D.E., Jang G.M., Bouhaddou M., Xu J., Obernier K., White K.M., O'Meara M.J., Rezelj V.V., Guo J.Z., Swaney D.L. (2020). A SARS-CoV-2 protein interaction map reveals targets for drug repurposing. Nature.

[15] Meyniel-Schicklin L., de Chassey B., André P., Lotteau V. (2012). Viruses and Interactomes in Translation. Mol. Cell. Proteomics.

[16] Shah P.S., Wojcechowskyj J.A., Eckhardt M., Krogan N.J. (2015). Comparative mapping of host–pathogen protein–protein interactions. Curr. Opin. Microbiol..

[17] Subramani C., Nair V.P., Anang S., Mandal S.D., Pareek M., Kaushik N., Srivastava A., Saha S., Shalimar, Nayak B. (2018). Host-Virus Protein Interaction Network Reveals the Involvement of Multiple Host Processes in the Life Cycle of Hepatitis E Virus. mSystems.

[18] Uetz P., Dong Y.A., Zeretzke C., Atzler C., Baiker A., Berger B., Rajagopala S.V., Roupelieva M., Rose D., Fossum E., Haas J. (2006). Herpesviral protein networks and their interaction with the human proteome. Science.

[19] Greco T.M., Diner B.A., Cristea I.M. (2014). The Impact of Mass Spectrometry–Based Proteomics on Fundamental Discoveries in Virology. Annu. Rev. Virol..

[20] Bouhaddou M., Memon D., Meyer B., White K.M., Rezelj V.V., Correa Marrero M., Polacco B.J., Melnyk J.E., Ulferts S., Kaake R.M. (2020). The Global Phosphorylation Landscape of SARS-CoV-2 Infection. Cell.

[21] Hashimoto Y., Sheng X., Murray-Nerger L.A., Cristea I.M. (2020). Temporal dynamics of protein complex formation and dissociation during human cytomegalovirus infection. Nat. Commun..

[22] Justice J.L., Kennedy M.A., Hutton J.E., Liu D., Song B., Phelan B., Cristea I.M. (2021). Systematic profiling of protein complex dynamics reveals DNA-PK phosphorylation of IFI16 en route to herpesvirus immunity. Sci. Adv..

[23] Gao J., Buldyrev S.V., Stanley H.E., Havlin S. (2012). Networks formed from interdependent networks. Nat. Phys..

[24] (2021). Networks of Networks in Biology: Concepts, Tools and Applications.

[25] Aguirre J., Papo D., Buldú J.M. (2013). Successful strategies for competing networks. Nat. Phys..

[26] Iranzo J., Buldú J.M., Aguirre J. (2016). Competition among networks highlights the power of the weak. Nat. Commun..

[27] Buldú J.M., Pablo-Martí F., Aguirre J. (2019). Taming out-of-equilibrium dynamics on interconnected networks. Nat. Commun..

[28] Manrubia S., Cuesta J.A., Aguirre J., Ahnert S.E., Altenberg L., Cano A.V., Catalán P., Diaz-Uriarte R., Elena S.F., García-Martín J.A. (2021). From genotypes to organisms: State-of-the-art and perspectives of a cornerstone in evolutionary dynamics. Phys. Life Rev..

[29] Newman M.E. (2018).

[30] Soh T.K., Davies C.T.R., Muenzner J., Hunter L.M., Barrow H.G., Connor V., Bouton C.R., Smith C., Emmott E., Antrobus R. (2020). Temporal Proteomic Analysis of Herpes Simplex Virus 1 Infection Reveals Cell-Surface Remodeling via pUL56-Mediated GOPC Degradation. Cell Rep..

[31] Weekes M.P., Tomasec P., Huttlin E.L., Fielding C.A., Nusinow D., Stanton R.J., Wang E.C.Y., Aicheler R., Murrell I., Wilkinson G.W.G. (2014). Quantitative Temporal Viromics: An Approach to Investigate Host-Pathogen Interaction. Cell.

[32] Lovell D., Pawlowsky-Glahn V., Egozcue J.J., Marguerat S., Bähler J. (2015). A Valid Alternative to Correlation for Relative Data. PLoS Comput. Biol..

[33] Erb I., Notredame C. (2016). How should we measure proportionality on relative gene expression data?. Theory Biosci.

[34] Skinnider M.A., Squair J.W., Foster L.J. (2019). Evaluating measures of association for single-cell transcriptomics. Nat. Methods.

[35] Newman M.E.J. (2006). Modularity and community structure in networks. Proc. Natl. Acad. Sci. USA.

[36] Jean Beltran P.M., Cristea I.M. (2014). The life cycle and pathogenesis of human cytomegalovirus infection: lessons from proteomics. Expert Rev. Proteomics.

[37] Sanchez V., Britt W. (2021). Human Cytomegalovirus Egress: Overcoming Barriers and Co-Opting Cellular Functions. Viruses.

[38] Nightingale K., Lin K.M., Ravenhill B.J., Davies C., Nobre L., Fielding C.A., Ruckova E., Fletcher-Etherington A., Soday L., Nichols H. (2018). High-Definition Analysis of Host Protein Stability during Human Cytomegalovirus Infection Reveals Antiviral Factors and Viral Evasion Mechanisms. Cell Host Microbe.

[39] Stern-Ginossar N., Weisburd B., Michalski A., Le V.T.K., Hein M.Y., Huang S.X., Ma M., Shen B., Qian S.B., Hengel H. (2012). Decoding Human Cytomegalovirus. Science.

[40] Buldyrev S.V., Parshani R., Paul G., Stanley H.E., Havlin S. (2010). Catastrophic cascade of failures in interdependent networks. Nature.

[41] Aguirre J., Sevilla-Escoboza R., Gutiérrez R., Papo D., Buldú J.M. (2014). Synchronization of Interconnected Networks: The Role of Connector Nodes. Phys. Rev. Lett..

[42] Donetti L., Hurtado P.I., Muñoz M.A. (2005). Entangled Networks, Synchronization, and Optimal Network Topology. Phys. Rev. Lett..

[43] Langville A.N., Meyer C.D. (2011). Google’s PageRank and Beyond.

[44] Senanayake U., Piraveenan M., Zomaya A. (2015). The Pagerank-Index: Going beyond Citation Counts in Quantifying Scientific Impact of Researchers. PLoS One.

[45] Aguirre J., Buldú J.M., Manrubia S.C. (2009). Evolutionary dynamics on networks of selectively neutral genotypes: Effects of topology and sequence stability. Phys. Rev. E.

[46] Capitán J.A., Aguirre J., Manrubia S. (2015). Dynamical community structure of populations evolving on genotype networks. Chaos, Solit. Fractals.

[47] Luo H. (2016). Interplay between the virus and the ubiquitin–proteasome system: molecular mechanism of viral pathogenesis. Curr. Opin. Virol..

[48] Zhu H., Zheng C. (2020). The Race between Host Antiviral Innate Immunity and the Immune Evasion Strategies of Herpes Simplex Virus 1. Microbiol. Mol. Biol. Rev..

[49] Rodríguez M.C., Dybas J.M., Hughes J., Weitzman M.D., Boutell C. (2020). The HSV-1 ubiquitin ligase ICP0: Modifying the cellular proteome to promote infection. Virus Res..

[50] Hobbs W.E., DeLuca N.A. (1999). Perturbation of Cell Cycle Progression and Cellular Gene Expression as a Function of Herpes Simplex Virus ICP0. J. Virol..

[51] Lanz T.V., Brewer R.C., Ho P.P., Moon J.S., Jude K.M., Fernandez D., Fernandes R.A., Gomez A.M., Nadj G.S., Bartley C.M. (2022). Clonally expanded B cells in multiple sclerosis bind EBV EBNA1 and GlialCAM. Nature.

[52] Saez-Calveras N., Stuve O. (2022). The role of the complement system in Multiple Sclerosis: A review. Front. Immunol..

[53] Gao Y., Wang L., Lei Z., Li J., Forrest J.C., Liang X. (2019). IRF4 promotes Epstein–Barr virus activation in Burkitt’s lymphoma cells. J. Gen. Virol..

[54] Shaffer A.L., Emre N.C.T., Romesser P.B., Staudt L.M. (2009). Immunity. Malignancy! Therapy?. Clin. Cancer Res..

[55] Pan X., Liu J.-H. (2021). Identification of four key biomarkers and small molecule drugs in nasopharyngeal carcinoma by weighted gene co-expression network analysis. Bioengineered.

[56] Dochi H., Kondo S., Murata T., Fukuyo M., Nanbo A., Wakae K., Jiang W.P., Hamabe-Horiike T., Tanaka M., Nishiuchi T. (2022). Estrogen induces the expression of EBV lytic protein ZEBRA, a marker of poor prognosis in nasopharyngeal carcinoma. Cancer Sci..

[57] Dong H.-J., Zhang R., Kuang Y., Wang X.-J. (2021). Selective regulation in ribosome biogenesis and protein production for efficient viral translation. Arch. Microbiol..

[58] Paladino P., Marcon E., Greenblatt J., Frappier L. (2014). Identification of Herpesvirus Proteins That Contribute to G1/S Arrest. J. Virol..

[59] Rozman B., Nachshon A., Levi Samia R., Lavi M., Schwartz M., Stern-Ginossar N. (2022). Temporal dynamics of HCMV gene expression in lytic and latent infections. Cell Rep..

[60] Sergi C.M., Gilmour S. (2022). Biliary Atresia: A Complex Hepatobiliary Disease with Variable Gene Involvement, Diagnostic Procedures, and Prognosis. Diagnostics.

[61] Zhao Y., Xu X., Liu G., Yang F., Zhan J. (2021). Prognosis of Biliary Atresia Associated With Cytomegalovirus: A Meta-Analysis. Front. Pediatr..

[62] Hertel L., Mocarski E.S. (2004). Global Analysis of Host Cell Gene Expression Late during Cytomegalovirus Infection Reveals Extensive Dysregulation of Cell Cycle Gene Expression and Induction of Pseudomitosis Independent of US28 Function. J. Virol..

[63] Oberstein A., Shenk T. (2017). Cellular responses to human cytomegalovirus infection: Induction of a mesenchymal-to-epithelial transition (MET) phenotype. Proc. Natl. Acad. Sci. USA.

[64] Dongre A., Weinberg R.A. (2019). New insights into the mechanisms of epithelial–mesenchymal transition and implications for cancer. Nat. Rev. Mol. Cell Biol..

[65] Greseth M.D., Traktman P. (2022). The Life Cycle of the Vaccinia Virus Genome. Annu. Rev. Virol..

[66] Liu L., Cooper T., Howley P.M., Hayball J.D. (2014). From Crescent to Mature Virion: Vaccinia Virus Assembly and Maturation. Viruses.

[67] Depierreux D.M., Altenburg A.F., Soday L., Fletcher-Etherington A., Antrobus R., Ferguson B.J., Weekes M.P., Smith G.L. (2022). Selective modulation of cell surface proteins during vaccinia infection: A resource for identifying viral immune evasion strategies. PLoS Pathog..

[68] Arvanitis D., Davy A. (2008). Eph/ephrin signaling: networks. Genes Dev..

[69] de Boer E.C.W., van Gils J.M., van Gils M.J. (2020). Ephrin-Eph signaling usage by a variety of viruses. Pharmacol. Res..

[70] Noren N.K., Pasquale E.B. (2004). Eph receptor–ephrin bidirectional signals that target Ras and Rho proteins. Cell. Signal..

[71] Sanderson C.M., Way M., Smith G.L. (1998). Virus-Induced Cell Motility. J. Virol..

[72] Chiu Y.-F., Sugden B. (2016). Epstein-Barr Virus: The Path from Latent to Productive Infection. Annu. Rev. Virol..

[73] Jäger S., Cimermancic P., Gulbahce N., Johnson J.R., McGovern K.E., Clarke S.C., Shales M., Mercenne G., Pache L., Li K. (2012). Global landscape of HIV–human protein complexes. Nature.

[74] Sugimoto A. (2022). Replication Compartments—The Great Survival Strategy for Epstein–Barr Virus Lytic Replication. Microorganisms.

[75] Meng X., Wei L., Peng X.P., Zhao X. (2019). Sumoylation of the DNA polymerase ε by the Smc5/6 complex contributes to DNA replication. PLoS Genet..

[76] Irwan I.D., Cullen B.R. (2023). The SMC5/6 complex: An emerging antiviral restriction factor that can silence episomal DNA. PLoS Pathog..

[77] de la Fuente A. (2010). From ‘differential expression’ to ‘differential networking’ – identification of dysfunctional regulatory networks in diseases. Trends Genet..

[78] Langfelder P., Horvath S. (2008). WGCNA: an R package for weighted correlation network analysis. BMC Bioinf..

[79] Saint-Antoine M.M., Singh A. (2020). Network inference in systems biology: recent developments, challenges, and applications. Curr. Opin. Biotechnol..

[80] Lant S., Maluquer de Motes C. (2021). Poxvirus Interactions with the Host Ubiquitin System. Pathogens.

[81] Sloan E., Tatham M.H., Groslambert M., Glass M., Orr A., Hay R.T., Everett R.D. (2015). Analysis of the SUMO2 Proteome during HSV-1 Infection. PLoS Pathog..

[82] Chen Y., Lun A.T.L., Smyth G.K. (2016). From reads to genes to pathways: differential expression analysis of RNA-Seq experiments using Rsubread and the edgeR quasi-likelihood pipeline. F1000Res..

[83] Gillespie C.S. (2015). Fitting Heavy Tailed Distributions: The poweRlaw Package. J. Stat. Softw..

[84] Csardi G., Nepusz T. (2006). The igraph software package for complex network research. InterJournal Complex Syst..

[85] Wu T., Hu E., Xu S., Chen M., Guo P., Dai Z., Feng T., Zhou L., Tang W., Zhan L. (2021). A universal enrichment tool for interpreting omics data. Innovation.

[86] Kolberg L., Raudvere U., Kuzmin I., Vilo J., Peterson H. (2020). gprofiler2 -- an R package for gene list functional enrichment analysis and namespace conversion toolset g:Profiler. F1000Res..

[87] Garcia-Moreno A., López-Domínguez R., Villatoro-García J.A., Ramirez-Mena A., Aparicio-Puerta E., Hackenberg M., Pascual-Montano A., Carmona-Saez P. (2022). Functional Enrichment Analysis of Regulatory Elements. Biomedicines.

[88] Golumbeanu M., Desfarges S., Hernandez C., Quadroni M., Rato S., Mohammadi P., Telenti A., Beerenwinkel N., Ciuffi A. (2019). Proteo-Transcriptomic Dynamics of Cellular Response to HIV-1 Infection. Sci. Rep..

[89] Robinson M.D., Oshlack A. (2010). A scaling normalization method for differential expression analysis of RNA-seq data. Genome Biol..

[90] Friedman J., Alm E.J. (2012). Inferring Correlation Networks from Genomic Survey Data. PLoS Comput. Biol..

[91] Lin L.I.-K. (1989). A Concordance Correlation Coefficient to Evaluate Reproducibility. Biometrics.

[92] Quinn T.P., Richardson M.F., Lovell D., Crowley T.M. (2017). An R-package for Identifying Proportionally Abundant Features Using Compositional Data Analysis. Sci. Rep..

[93] Traag V.A., Waltman L., van Eck N.J. (2019). From Louvain to Leiden: guaranteeing well-connected communities. Sci. Rep..

