# Supplementary material for: Virus-host protein co-expression networks reveal temporal organization and strategies of viral infection
Source: iScience. 2023 Nov 16;26(12):108475. doi: 10.1016/j.isci.2023.108475 (PMC10698274; doi:10.1016/j.isci.2023.108475)
Supplement: Document S1. Figures S1–S4 and Tables S1–S3 [file mmc1.pdf]

iScience, Volume *26*

Supplemental information

Virus-host protein co-expression networks reveal temporal organization and strategies of viral infection

Jacobo Aguirre and Raúl Guantes

	HERPES VIRUSES			POX VIRUS
	**HSV-1**	**EBV**	**HCMV**	**VACV**
**Genome size**	~152 kbp, > 200 ORFs[1]	~170 kbp, ~100 ORFs[2]	~235 kbp, ~500 ORFs[3]	~190 kbp, >200 ORFs[4]
**Host cell line**	HaCat (human keratynocite)	P3HR1(Burkitt lymphoma)	HFFF (primary human fetal foreskin fibroblasts)	HFFF (primary human fetal foreskin fibroblasts)
**Reference**	Soh et al. 2020[5]	Ersing et al. 2017[6]	Nightingale et al. 2018[7]	Soday et al. 2019[8]
**# time points**	7 (0, 2, 4, 6, 9, 12 and 18h pi)	5(0, 15, 24, 48 and 72h pi)	8(0, 6, 12, 18, 24, 48, 72 and 96h pi)	7 (0, 2, 4, 6, 8, 12 and 18h pi)
**# viral proteins quantified***	75	64	124	164
**# host proteins quantified***	6,875	6,791	7,635	8,271
**# viral proteins differentially expressed (FDR< 0.05)#**	74	63	119	139
**# host proteins differentially expressed (FDR < 0.05)#**	1,261	1,209	808	967
**Immediate early genes/proteins&**	*R_L2*/ICP0, R_S1/ICP4, U_S1/ICP22, U_L54/ICP27[9]	*BZLF1, BRLF1*[10]	*UL103, UL104, UL115,UL119*[11]	*B11, A48, F11, K1*[8]

Table S1. Information on the viruses and experimental data re-analyzed in this work, **related to Figures 1 and 2**.

*We analyzed only proteins quantified at all time points in biological replicates.

#Abundance data expressed as pseudo-counts were analyzed for differential expression with respect to the control (non-infected or non-lytic samples) using the *edgeR* pipeline[12] and a paired design, controlling for differences between biological replicates (STAR Methods). We include proteins differentially expressed with a false detection rate (FDR) < 0.05 at any time point during the course of infection.

&Documented immediate early genes of each virus present in the reconstructed co-expression networks and used for analyses in this work.

	HERPES VIRUSES			POX VIRUS
	**HSV-1**	**EBV**	**HCMV**	**VACV**
**Absolute fold-change cutoff for number of nodes**	2	2	3	1.5
**Concordance coefficient cutoff for number of edges**	0.97	0.95	0.97	0.97
**Number of viral/host nodes giant component**	71/400	59/495	76/452	134/288
**Number of edges giant component**	4,430	4,840	4,521	5,323
**Scale-free[$]**	No	No	No	No
**# Communities**	8	6	5	5
**Modularity coefficient[#]**	0.61	0.71	0.63	0.66
**# up/down-regulated nodes**	74/394	278/263	144/360	137/285
**#positive/negative links**	4,275/139	3,160/1,165	3,978/487	5,305/18

Table S2. Parameters and properties of reconstructed virus-host protein co-expression networks, related to Figures 1 and 2.

[$]The scale-free property is assessed by the goodness-of-fit test of the degree distribution to a power law, using a maximum likelihood procedure as described in Clauset et al. (2009)[13] and implemented in the *R* package *poweRlaw*[14].

[#]The modularity coefficient is a measure of the difference between the actual number of edges within communities, and the expected number of edges if they were placed at random, as defined in Clauset et al. (2004)[15].

VACV	# Virus/Host Nodes	Mean deg.	% eigenc.	λ_1	λ_2	λ_1/λ_2	# Up/Down nodes	# +/- links
**Com. 1**	71/0	24.1	1.8e-7	31.47	16.92	1.86	71/0	854/0
**Com. 2**	62/0	30.9	2.5e-6	35.97	14.3	2.51	62/0	959/0
**Com. 3**	1/13	5.6	0.03	6.74	3.21	2.10	1/13	36/3
**Com. 4**	0/141	24.8	30.2	35.90	20.83	1.72	2/139	1,738/12
**Com. 5**	0/134	19.9	69.8	31.25	18.71	1.67	1/133	1,334/2
**Viral net**	134	28.5	3.2e-6	36.44	31.65	1.15	134/0	1,910/0
**Host net**	288	23.7	100	38.01	31.69	1.2	3/285	3,395/14
**EBV**	**# Virus/Host Nodes**	**Mean deg.**	**% eigenc.**	**λ_1**	**λ_2**	**λ_1/λ_2**	**# Up/Down nodes**	**# +/- links**
**Com. 1**	59/0	30.0	94.1	35.68	11.25	3.17	59/0	885/0
**Com. 2**	0/13	2.5	5.9	3.16	2.37	1.33	11/2	9/7
**Com. 3**	0/143	10.4	5.7e-3	16.60	12.92	1.28	52/91	466/277
**Com. 4**	0/117	14.1	0.033	25.55	12.74	2.01	71/46	416/407
**Com. 5**	0/107	21.6	3.3e-3	28.92	20.51	12.36	35/72	715/438
**Com. 6**	0/102	14.7	1.9e-5	23.40	14.96	1.56	50/52	401/349
**Viral net**	59	30.0	94.1	35.68	11.25	3.17	59/0	885/0
**Host net**	482	15.9	5.9	31.47	25.97	1.21	219/263	2,192/1,633
**HCMV**	**# Virus/Host Nodes**	**Mean deg.**	**% eigenc.**	**λ_1**	**λ_2**	**λ_1/λ_2**	**# Up/Down nodes**	**# +/- links**
**Com. 1**	59/52	12.7	0.3	21.24	13.57	1.57	87/24	607/98
**Com. 2**	11/83	12.6	7.4	20.45	10.45	1.96	30/64	427/164
**Com. 3**	2/109	12.5	4.9	19.97	13.22	1.51	22/89	591/105
**Com. 4**	0/73	16.4	32.8	24.23	9.82	2.47	4/69	558/42
**Com. 5**	0/115	19.8	54.4	28.84	19.41	1.49	1/114	1,1130/7
**Viral net**	72	8.9	0.3	16.73	8.55	1.96	72/0	319/0
**Host net**	432	17.6	99.7	32.27	27.11	1.19	72/360	3,428/384
**HSV-1**	**# Virus/Host Nodes**	**Mean deg.**	**% eigenc.**	**λ_1**	**λ_2**	**λ_1/λ_2**	**# Up/Down nodes**	**# +/- links**
**Com. 1**	68/0	11.4	0.002	16.88	12.31	1.37	68/0	389/0
**Com. 2**	0/25	3.4	0.003	5.06	3.31	1.53	0/25	42/0
**Com. 3**	0/76	15.0	1.9	19.15	14.47	1.32	1/75	563/7
**Com. 4**	0/56	16.4	11.9	21.53	9.78	2.20	0/56	458/0
**Com. 5**	0/62	16.8	19.8	25.06	8.04	3.11	4/58	518/3
**Com. 6**	0/74	20.3	42.0	27.86	13.96	2.0	1/73	750/1
**Com. 7**	0/20	5.9	0.2	7.63	4.15	1.84	0/20	59/0
**Com. 8**	0/87	14.1	24.2	22.45	12.48	1.80	0/87	615/0
**Viral net**	68	11.4	0.002	16.88	12.31	1.37	68/0	389/0
**Host net**	400	19.5	99.9	33.65	30.09	1.12	6/394	3,886/11

Table S3. Properties of the different communities and viral/host subnetworks for each virus analyzed, related to Figure 3. Columns correspond to viral/host nodes per community; mean degree of each community; percentage of eigenvector centrality obtained by community; first and second largest eigenvalues of the adjacency matrix and their ratio; number of upregulated/downregulated nodes per community; number of positive/negative links.

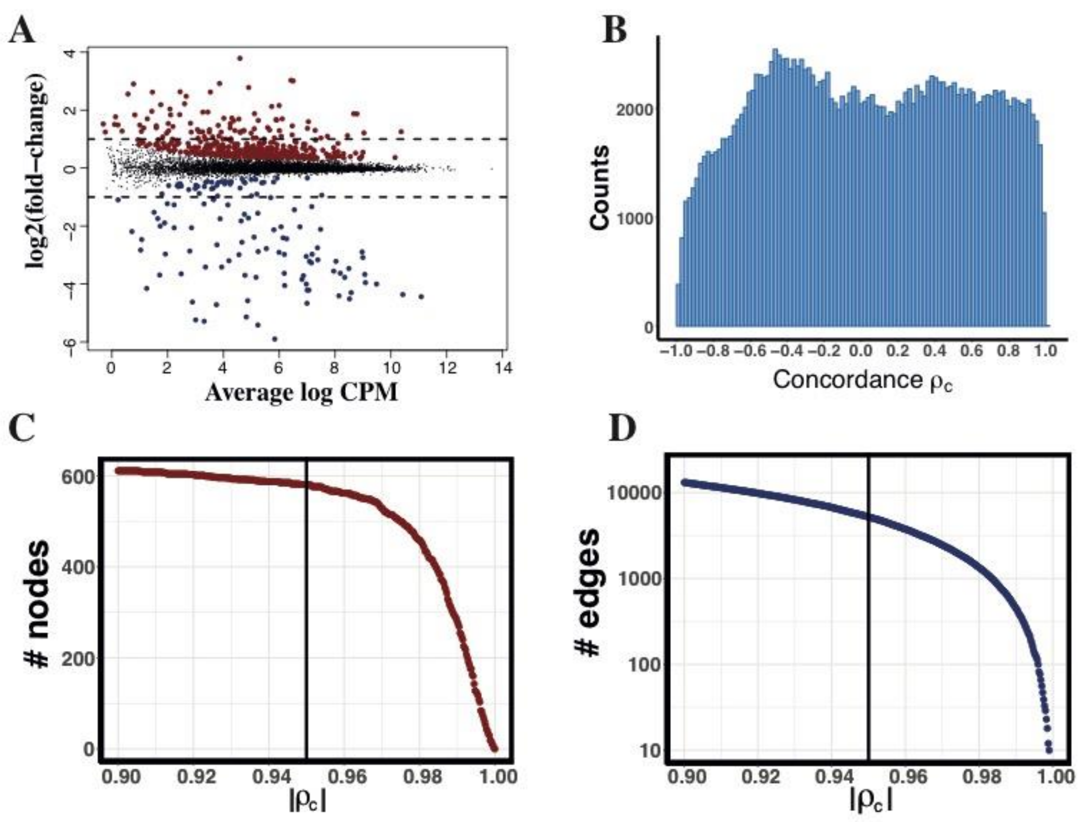

Figure S1. Choice of cut-off values for network reconstruction. A. Abundance of each protein (expressed as average over replicates in counts-per-million (CPM)) versus base 2 log of fold-change (relative to uninfected/control samples). Red/blue dots are up/down regulated proteins with FDR < 0.05 (STAR Methods). Dashed lines indicate the cut-off value in fold-change to select relevant nodes. This example corresponds to HSV-1 at 4h post-infection. B. Distribution of concordance coefficients ρ_c between all selected nodes for the EBV (containing comparable numbers of positive and negative concordances, Table S3). Coefficients are roughly homogeneously distributed between [-0.6,0.9], but frequencies abruptly decrease for $|\rho_c|>0.9$. C. Number of nodes that are left in the network as a function of $|\rho_c|$ for the EBV. D. Number of edges left in the network as a function of $|\rho_c|$ for the EBV. Vertical lines indicate the cut-off value chosen for $|\rho_c|$ giving a good trade-off between number of nodes/edges to avoid too dense or too sparse/disconnected network.

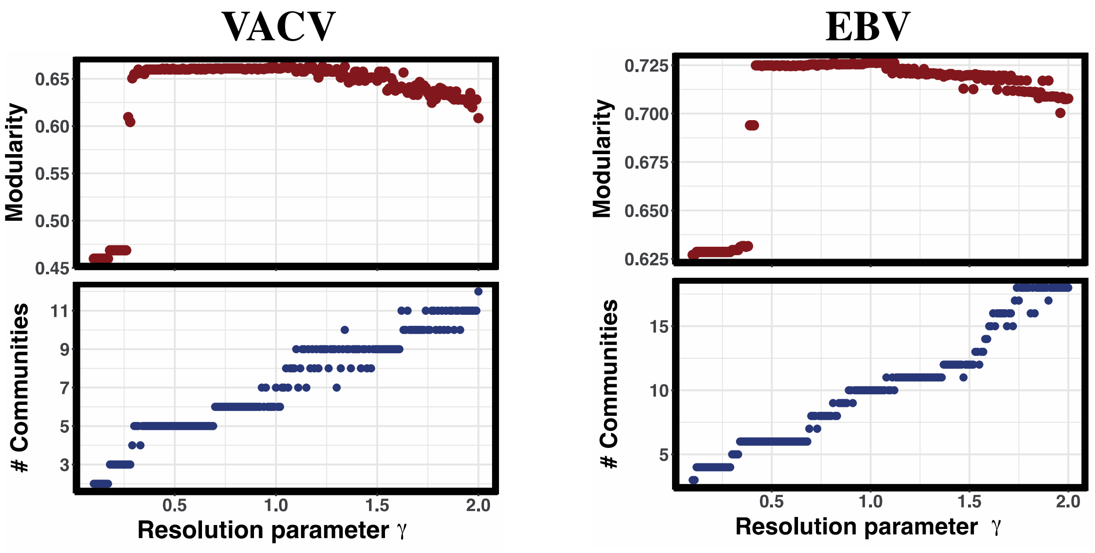

Figure S2. Selection of resolution parameter for community partition with Leiden algorithm. Optimized modularity coefficient (top panels) and number of different communities (bottom panels) as a function of the resolution parameter γ. Taking VACV and EBV virus-host protein co-expression networks as an example, we see that the plateaus in number of communities around γ=0.5 coincide with a sudden increase in modularity. By inspection of the contingency tables of community memberships of different partitions, we see that plateaus at larger γ values are the result of segregation of few nodes (< 10) from large communities (for instance, the plateau around γ=0.8 in VACV just adds a new community of 3 nodes detached from a large community of 134 nodes) or the splitting of a large community into two. In both cases, modularity worsens and partitions are less robust (as the plateaus around γ=1.3 in VACV). We thus choose γ=0.5 as the resolution parameter for these two examples, giving both optimal and more parsimonious and robust partitions.

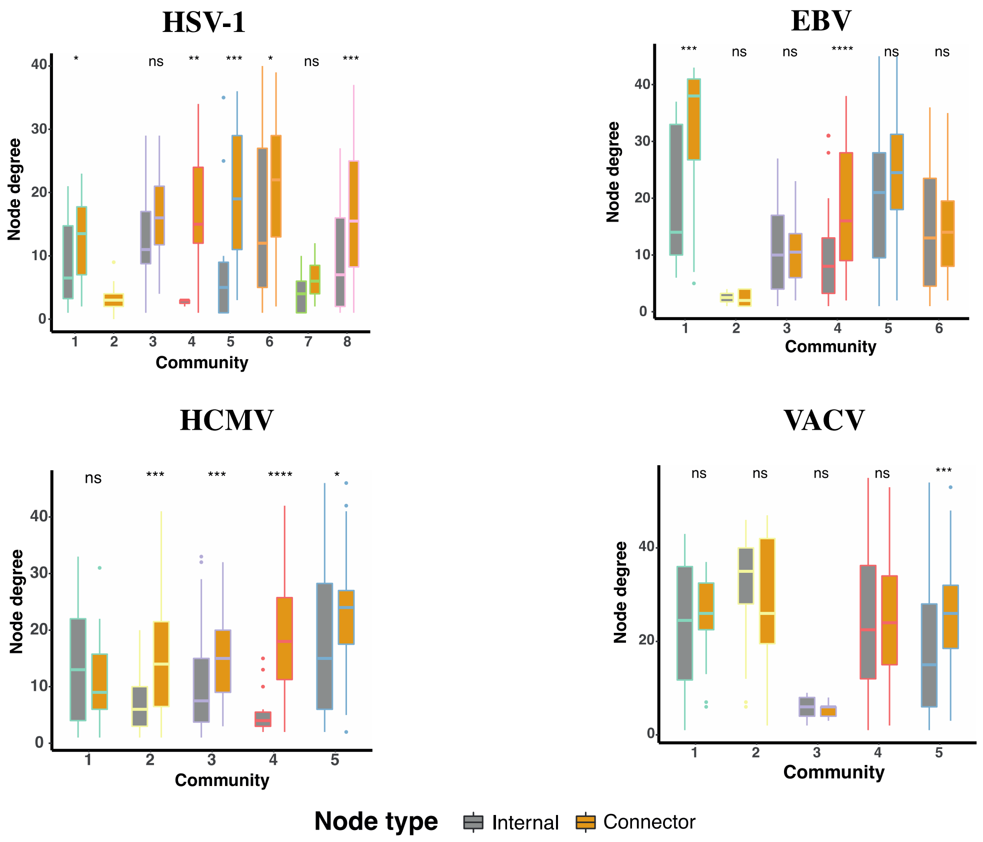

Figure S3. Degree of internal and connector nodes.
Distributions of node degrees for each virus type and community, separating internal nodes (nodes connected only to other members of the same community, gray boxes) from connector nodes (nodes connecting to other communities, orange boxes). Significance p-values for difference of the mean between internal and connector nodes are calculated with Wilcoxon test (ns: $p>0.05$, *: $p \leq 0.05$, ** : $p \leq 0.01$, *** : $p \leq 0.001$, **** : $p \leq 0.0001$).

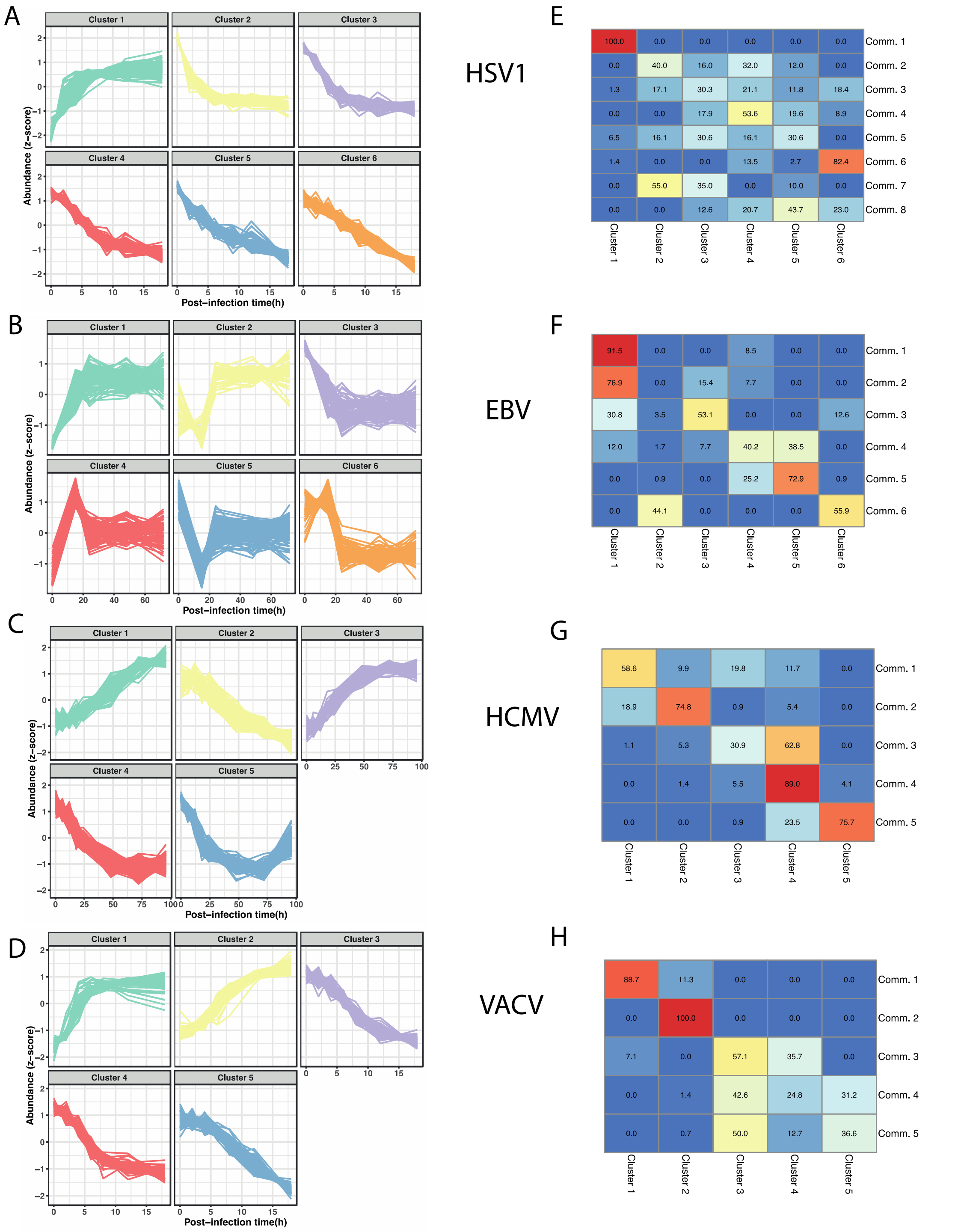

Figure S4. Clustering of temporal profiles. Temporal profiles of all network nodes were clustered by similarity using a soft-clustering method employing mixed-effects models and spline fitting[16](‘*TMixClust*’ package in R/Bioconductor). Analysis of silouhette plots[17] showed that partitions in 5-6 temporal classes were optimal. A-D: Time series of protein abundances (z-score normalized) grouped by similarity profiles using the clustering method. E-H: Heatmap representation of the contingency table between clustering and community partitions. The numbers represent the percentages of proteins present both in the specified cluster and community.

REFERENCES

1. Whisnant, A.W., Jürges, C.S., Hennig, T., Wyler, E., Prusty, B., Rutkowski, A.J., L'hernault, A., Djakovic, L., Göbel, M., Döring, K., et al. (2020). Integrative functional genomics decodes herpes simplex virus 1. Nat. Commun. *11*, 2038. 10.1038/s41467-020-15992-5.

2. Wegner, F., Lassalle, F., Depledge, D.P., Balloux, F., and Breuer, J. (2019). Coevolution of Sites under Immune Selection Shapes Epstein–Barr Virus Population Structure. Mol. Biol. Evol. *36*, 2512–2521. 10.1093/molbev/msz152.

3. Erhard, F., Halenius, A., Zimmermann, C., L'Hernault, A., Kowalewski, D.J., Weekes, M.P., Stevanovic, S., Zimmer, R., and Dölken, L. (2018). Improved Ribo-seq enables identification of cryptic translation events. Nat. Methods *15*, 363–366. 10.1038/nmeth.4631.

4. Tombácz, D., Prazsák, I., Csabai, Z., Moldován, N., Dénes, B., Snyder, M., and Boldogkői, Z. (2020). Long-read assays shed new light on the transcriptome complexity of a viral pathogen. Sci. Rep. *10*, 13822. 10.1038/s41598-020-70794-5.

5. Soh, T.K., Davies, C.T.R., Muenzner, J., Hunter, L.M., Barrow, H.G., Connor, V., Bouton, C.R., Smith, C., Emmott, E., Antrobus, R., et al. (2020). Temporal Proteomic Analysis of Herpes Simplex Virus 1 Infection Reveals Cell-Surface Remodeling via pUL56-Mediated GOPC Degradation. Cell Rep. *33*. 10.1016/j.celrep.2020.108235.

6. Ersing, I., Nobre, L., Wang, L.W., Soday, L., Ma, Y., Paulo, J.A., Narita, Y., Ashbaugh, C.W., Jiang, C., Grayson, N.E., et al. (2017). A Temporal Proteomic Map of Epstein-Barr Virus Lytic Replication in B Cells. Cell Rep. *19*, 1479–1493. 10.1016/j.celrep.2017.04.062.

7. Nightingale, K., Lin, K.-M., Ravenhill, B.J., Davies, C., Nobre, L., Fielding, C.A., Ruckova, E., Fletcher-Etherington, A., Soday, L., Nichols, H., et al. (2018). High-Definition Analysis of Host Protein Stability during Human Cytomegalovirus Infection Reveals Antiviral Factors and Viral Evasion Mechanisms. Cell Host Microbe *24*, 447-460.e11. 10.1016/j.chom.2018.07.011.

8. Soday, L., Lu, Y., Albarnaz, J.D., Davies, C.T.R., Antrobus, R., Smith, G.L., and Weekes, M.P. (2019). Quantitative Temporal Proteomic Analysis of Vaccinia Virus Infection Reveals Regulation of Histone Deacetylases by an Interferon Antagonist. Cell Rep. *27*, 1920-1933.e7. 10.1016/j.celrep.2019.04.042.

9. Ibáñez, F.J., Farías, M.A., Gonzalez-Troncoso, M.P., Corrales, N., Duarte, L.F., Retamal-Díaz, A., and González, P.A. (2018). Experimental Dissection of the Lytic Replication Cycles of Herpes Simplex Viruses in vitro. Front. Microbiol. *9*.

10. Miller, G., El-Guindy, A., Countryman, J., Ye, J., and Gradoville, L. (2007). Lytic Cycle Switches of Oncogenic Human Gammaherpesviruses1. In Advances in Cancer Research (Academic Press), pp. 81–109. 10.1016/S0065-230X(06)97004-3.

11. Rozman, B., Nachshon, A., Samia, R.L., Lavi, M., Schwartz, M., and Stern-Ginossar, N. (2022). Temporal dynamics of HCMV gene expression in lytic and latent infections. Cell Rep. *39*. 10.1016/j.celrep.2022.110653.

12. Chen, Y., Lun, A.T.L., and Smyth, G.K. (2016). From reads to genes to pathways: differential expression analysis of RNA-Seq experiments using Rsubread and the edgeR quasi-likelihood pipeline. Preprint, 10.12688/f1000research.8987.2 10.12688/f1000research.8987.2.

13. Clauset, A., Shalizi, C.R., and Newman, M.E.J. (2009). Power-Law Distributions in Empirical Data. SIAM Rev. *51*, 661–703. 10.1137/070710111.

14. Gillespie, C.S. (2015). Fitting Heavy Tailed Distributions: The poweRlaw Package. J. Stat. Softw. *64*, 1–16. 10.18637/jss.v064.i02.

15. Clauset, A., Newman, M.E.J., and Moore, C. (2004). Finding community structure in very large networks. Phys. Rev. E *70*, 066111. 10.1103/PhysRevE.70.066111.

16. Golumbeanu, M., Desfarges, S., Hernandez, C., Quadroni, M., Rato, S., Mohammadi, P., Telenti, A., Beerenwinkel, N., and Ciuffi, A. (2019). Proteo-Transcriptomic Dynamics of Cellular Response to HIV-1 Infection. Sci. Rep. *9*, 213. 10.1038/s41598-018-36135-3.

17. Rousseeuw, P.J. (1987). Silhouettes: A graphical aid to the interpretation and validation of cluster analysis. J. Comput. Appl. Math. *20*, 53–65. 10.1016/0377-0427(87)90125-7.
